# Alcoholic Stimulants in Snake-Bites

**Published:** 1858-08

**Authors:** 


### Alcoholic Stimulants in Snake-bites.

From a letter received from Dr. John J. Addy, of Camden, Ark., we learn that upon the Western Frontier, where Rattle-Snakes are numerous, and where physicians are frequently called to treat poisonous wounds inflicted by this and other poisonous reptiles, they rely almost exclusively upon the administration of alcoholic stimulants, and in quantities sufficient to produce intoxication. It would appear from his letter, that the patient is not considered safe until drunkenness is induced—that they regard drunkenness, under such circumstances, as an evidence that the effects of the poison has been overcome by the stimulant.

Dr. Addy mentions a case that he had treated with perfect success by the free use of Whisky—the patient being perfectly relieved in twenty-four hours after the reception of the injury.

